# Expression and prognostic role of E2F transcription factors in high‐grade glioma

**DOI:** 10.1111/cns.13295

**Published:** 2020-02-16

**Authors:** Hai Yu, Zhijin Li, Maode Wang

**Affiliations:** ^1^ Department of Neurosurgery The First Affiliated Hospital of Xi'an Jiaotong University Xi'an China

**Keywords:** E2F7, E2F8, E2Fs, HGG, outcome, radioresistance

## Abstract

**Introduction:**

Patients with high‐grade glioma (HGG) suffered poor survival due to inherent or acquired therapeutic resistance and refractory recurrence. The outcome of HGG patients has improved little during the past decade. Therefore, molecular signatures are urgently needed for improving diagnosis, survival prediction and identification of therapeutic targets for HGG. E2F transcription factors (E2Fs), a family of transcription factors recognized as master regulators of cell proliferation, have been found to be involved in the pathogenesis of various tumor types.

**Aims:**

To investigate the expression of E2Fs and their prognosis value in high‐grade glioma (HGG).

**Results:**

Expression of E2Fs was analyzed in 394 HGG samples from TCGA dataset. E2Fs were generally expressed in HGG. Except for E2F3 and E2F5, expression of E2Fs was significantly upregulated and linked with grade progression. E2F1, E2F2, E2F7, and E2F8 were highly correlated with aggressive proliferation oncogenes, as well as potential therapeutic resistance oncogenes. Elevated E2Fs (not E2F3) were associated with adverse tumor features and poorer outcome. E2F7 and E2F8 exhibited superior outcome prediction performance compared with other E2Fs. Additionally, E2F7 and E2F8 independently predicted poorer survival in HGG patients. Gene set enrichment analysis identified a variety of critical oncogenic pathways that were tightly associated with E2F7 or E2F8, including epithelial‐mesenchymal transition, NFκB, STAT3, angiogenesis pathways. Furthermore, elevated expression of E2F7 indicated worse therapeutic response of HGG to irradiation and silencing of E2F7 conferred higher cell‐killing effect when combined with irradiation treatment. Mechanically, E2F7 directly regulates the transcriptional activity of EZH2 via binding at the corresponding promoter area.

**Conclusions:**

E2Fs (except for E2F3 and E2F5) are highly expressed in HGG and indicate adverse outcome. E2F7 and E2F8 were identified as novel potential prognostic markers in HGG. E2F7 was further validated to be closely associated with radioresistance of HGG and a critical transcriptional regulator of EZH2.

## 1 INTRODUCTION

Glioma is one of the most frequent and malignant primary brain tumors, accounting for more than 70% of adult brain tumors.^1^ High‐grade glioma (HGG) comprises anaplastic glioma and glioblastoma.^2^ The hallmarks of HGG are composed of aggressive infiltrative pattern, high proliferation rate, therapeutic resistance, and fast recurrence speed. The current available clinical treatment strategy includes maximum surgical resection, radiotherapy (IR), and temozolomide (TMZ) chemotherapy,^2^ while the survival outcome of HGG patients has improved little, owing the therapeutic resistance of HGG.^3^ Current study shows that pathological classification is limited, and molecular signatures are urgently needed for improving diagnosis and survival prediction for HGG.^4^

E2F transcription factors (E2Fs) are a family of members that play crucial role in coordinating the balance of cell cycle via a transcriptional axis.^5^ E2Fs can be generally sorted into three groups based on their structure and identified function,^6^ activators (E2F1‐3), canonical repressors (E2F4‐6), and atypical repressors (E2F7‐8). The expression of activators increases at the G1‐S phase transition, while the atypical repressors peak in the late S phase. Other members are generally expressed during all phases.^5^ The DNA binding ability of activators and canonical repressors is mediated by the protein complex with a member of the transcription factor dimerization partner family (TFDP1, TFDP2, or TFDP3).^7^ However, E2F7 and E2F8 are quite unique and they can bind E2F consensus sequences independent of the dimerization proteins.^8^, ^9^ Currently, multiple types of regulation of E2Fs have been identified. Transcriptionally, E2Fs are largely self‐regulated,^10^ and studies found that E2Fs’ subcellular localization was controlled by a variety of factors, like CRM1.^11^, ^12^, ^13^ In addition, a series of modifications to E2Fs have been identified, affecting the function, location, or stability of E2F family.^14^, ^15^

Accumulating evidence has implied that E2Fs were closely linked with tumorigenesis in a variety of cancer types.^16^, ^17^, ^18^ The elevated expression of E2Fs was found to be closely associated with poor prognosis of cancer patients, like pancreatic tumors.^19^ Among the E2Fs, E2F1 is the most widely investigated member in a huge panel of cancers. However, interestingly, in contrary to the previous defined function groups, some studies revealed that the atypical repressors also played a critical role in conferring aggressiveness to tumor cells. For example, Qing et al found that E2F8 transcriptionally promoted the expression of CCND1 via completely binding at the corresponding promoter area.^18^ This raise a possibility that the function of E2Fs in tumor cells might be hijacked by oncogenic factor and confer to distinct or opposite function as we current know. Studies revealed that the oncogenic role of E2Fs was not confined to aggressive cell cycle. Weijts et al found that E2F7 and E2F8 induced angiogenesis by interacting with hypoxia‐inducible factor 1 (HIF1) and promoting the expression of vascular endothelial growth factor A (VEGFA),^20^ two critical factors in HGG. In addition, E2F1 and E2F2 were reported to be highly associated with angiogenesis genes and cell interaction in breast cancer, conferring a more invasive phenotype.^21^ Interestingly, the study found that E2F1 regulated the glycolysis process in cancer cells, another hallmark of HGG.^22^ Via recruiting histone deacetylase and chromatin regulators, E2F7 promoted the DNA repair process in a transcription‐independent manner.^23^ However, even though the E2Fs have been reported to tightly linked with malignant behavior of multiple cancer cells, the expression pattern and prognosis role of E2Fs have not been fully characterized in HGG.

In the present study, we investigated the expression profile of E2Fs, as well as the survival outcome in HGG patients via the analysis of TCGA dataset. Gene set enrichment analysis was utilized to identify alternated oncogenic pathways. The therapeutic resistance role of E2Fs was explored, and we further validated the critical role of E2F7 in promoting radioresistance.

## 2 MATERIALS AND METHODS

### 2.1 Ethics

In this study, the usage of cell lines and experimental animals (nude mice) was approved by the Scientific Ethics Committee of Xi'an Jiaotong University, Xi'an, China.

### 2.2 Reagents

Following cell culture reagents were used: DMEM‐F12, Fetal bovine serum (FBS), Penicillin‐Streptomycin, Trypsin‐EDTA (Thermo scientific). Anti–β‐Actin (3700, Cell signaling, WB), anti‐E2F7 (Santa cruze, sc‐66870, WB, ChIP), anti‐rabbit IgG‐Horseradish peroxidase (NA934V), anti‐mouse IgG‐Horseradish peroxidase (NXA931) (GE Healthcare).

### 2.3 In vitro cell cultures

Glioma cell lines (U87 and U373) were provided by Xi'an Jiaotong University. Cell lines were cultivated in DMEM/F12 medium containing 10% FBS supplement (vol%), 1% Penicillin‐Streptomycin solution and the culture medium was changed every 2‐5 days.

### 2.4 RNA isolation and quantitative real‐time PCR

mRNA was isolated by Trizol (Thermo scientific) according to the manufacturer's protocol. cDNA was synthesized by using iScript reverse transcription supermix (Bio‐Rad) according to the manufacturer's protocol. qRT‐PCR was performed on StepOnePlus thermal cycler with SYBR Select Master Mix (Thermo scientific). Cycling conditions were 95°C for 5 minutes, and then 50 cycles of 95°C for 30 seconds, 60°C for 30 seconds, and 72°C 30 seconds. The primer sequences used in this study include the following:

- E2F7 (forward AGGCAGCCCAGACTAGATTTT; reverse GCTGGCAGCACTAATGAGCA)
- EZH2 (forward: AATCAGAGTACATGCGACTGAGA; reverse: GCTGTATCCTTCGCTGTTTCC)
- GAPDH (forward: GGAGCGAGATCCCTCCAAAAT; reverse: GGCTGTTGTCATACTTCTCATGG)

### 2.5 Cell viability assay

Viability of tumor cells was determined using AlamarBlue reagent (Thermo scientific). Cells were seeded at 1000 cells per well in a 96‐well plates, after indicated period of time AlamarBlue reagent was added into each well and 6 hours later fluorescence was measured (Excitation 515‐565 nm, Emission 570‐610 nm) using Synergy HTX multi‐mode reader (BioTek).

### 2.6 In vivo intracranial xenograft tumor models

About 6‐8 weeks old nude mice were used. The tumor cells suspension (1 × 10^5^ cells in 5 μL of PBS) was injected into the brains of mouse. When neuropathological symptoms developed, mice were sacrificed.

### 2.7 In vivo bioluminescent imaging

GBM tumor cells were transduced with lentiviral particles (pHAGE PGK‐GFP‐IRES‐LUC‐W) for coexpression of GFP and luciferase. Animals were administrated intraperitoneally with 2.5 mg/100 µL solution of XenoLight D‐luciferin (PerkinElmer) and anesthetized with isoflurane for the imaging analysis. The luciferase images were captured by using an IVIS 100 imaging system (PerkinElmer).

### 2.8 Lentivirus production and transduction

HEK293FT cells were transfected with the vectors (Sigma) and two packaging plasmids psPAX2 and pMGD2) using the CalPhos Mammalian Transfection Kit (Clontech) according to the manufacturer's protocol. HGG tumor cells were incubated with viral supernatants for 24 hours in the presence of 8 µg/mL polybrene.

Sequence of used shRNA: E2F7(TRCN0000017455, NM_203394.1‐2228s1c1 CCGGGCAACAGCAAACTCTCTTGTTCTCGAGAACAAGAGAGTTTGCTGTTGCTTTTT).

### 2.9 Chromatin immunoprecipitation

Chromatin immunoprecipitation was performed according to the manufacturer's protocol. Bioruptor UCD‐200 was used for sonication of DNA, and 200 000 cells were applied for following each reaction. Promoter sequence (EZH2): forward: CTGCACACCGCCTTCCT, reverse: CCGCCGTCTCTTTGTTCTT.

### 2.10 Gene expression data analysis

The data of publicly available datasets were download fromhttp://gliovis.bioinfo.cnio.es/.^24^ Gene Set Enrichment Analysis (GSEA) was performed using available online software (http://software.broadinstitute.org/gsea/index.jsp). Gene ontology analysis and KEGG analysis were performed using available online software (https://david-d.ncifcrf.gov/).

### 2.11 Statistical analysis

All data are presented as mean ± SD. Statistical differences between two groups were evaluated by two‐tailed *t* test. The comparison among multiple groups was performed by one‐way ANOVA analysis of variance followed by Tukey's post‐test. Statistical correlation was performed to calculate the regression R^2^ value and Pearson's correlation coefficient. The statistical significance of Kaplan‐Meier survival plot was determined by log‐rank analysis. For ROC analysis, all patients were divided into 2 groups based on the survival outcome. Area under curve (AUC) was used to evaluate the specificity and sensitivity. To analyze potential relationships between E2F7 and clinicopathologic features, logistic regression tests were used. The independent factors of survival were identified using Cox's proportional hazards model. Statistical analysis was performed by SPSS 22.0 or Prism 6 (GraphPad software). *P* < .05 was considered as statistically significant.

## 3 RESULTS

### 3.1 mRNA expression dynamics of E2Fs in HGG

To determine the expression profile of E2Fs in HGG, we performed data mining and analyzed the publicly available dataset, TCGA dataset.^25^ A total 394 cases (Table 1) were available for gene expression and clinical information, including 244 grade III and 150 grade IV cases. About 59.4% (n = 234) patients are male, and 39.1% (n = 154) patients are female. A total of 391 cases have been tested for the IDH1 mutation status. With respect to methylation status of MGMT promoter, 63.2% (n = 249) patients showed methylation of MGMT promoter and 28.9% (n = 114) cases were unmethylated, with 31 cases undetermined.

**Table 1:** Clinical characteristics of HGG patients

	Patient number	Percentage
Age		
≤50	173	43.9
>50	215	54.6
Missing	6	1.5
Gender		
Male	234	59.4
Female	154	39.1
Missing	6	1.5
Vital status		
Alive	205	52.0
Dead	188	47.7
Missing	1	0.3
WHO grade		
III	244	61.9
IV	150	38.1
IDH status		
Wide type	203	51.5
Mutant	188	47.7
Missing	3	0.8
MGMT promoter status		
Methylated	249	63.2
Unmethylated	114	28.9
Missing	31	7.9

**Figure 1:**
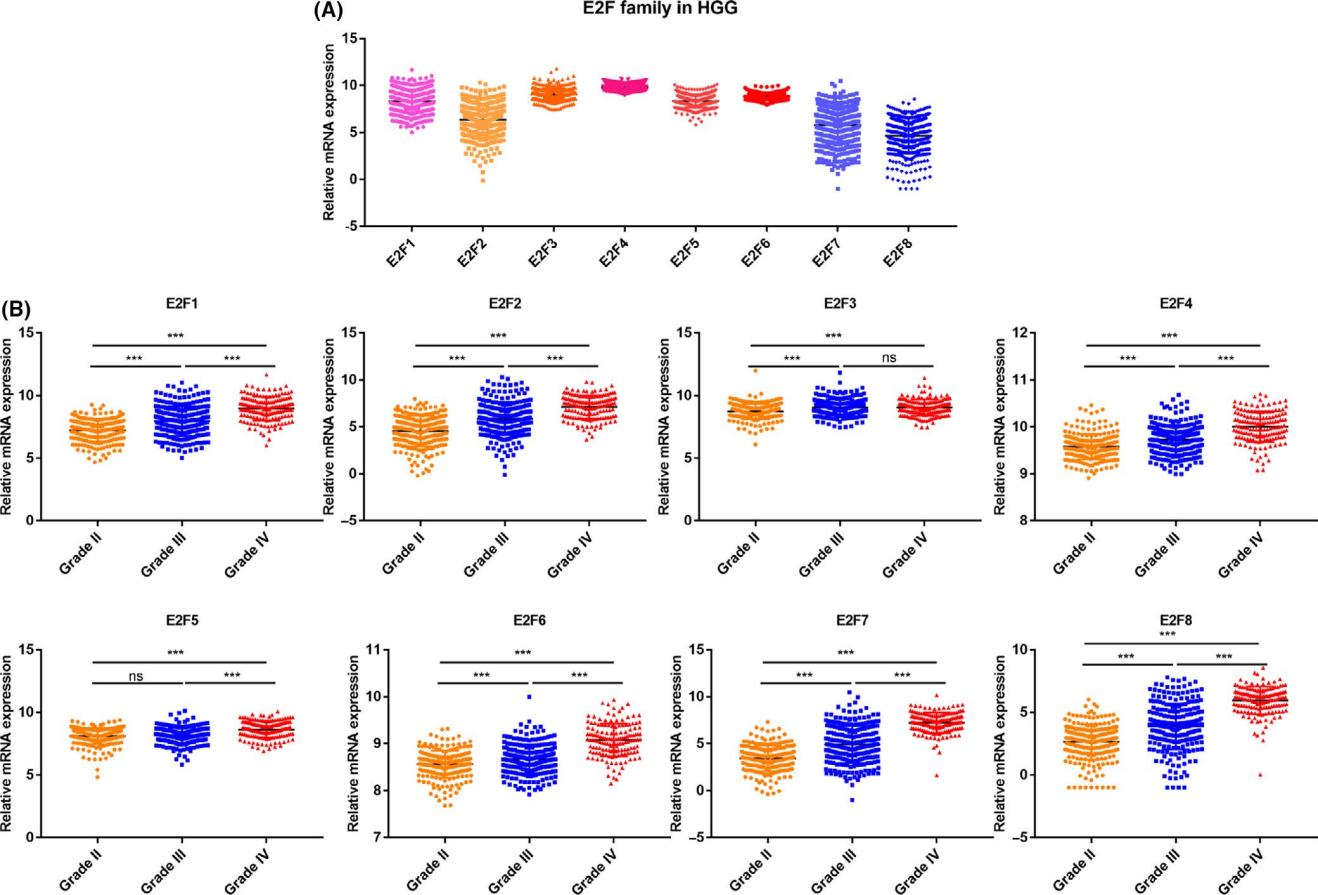
mRNA expression dynamics of E2Fs in HGG. A, Analysis of mRNA expression of E2Fs in HGG samples from TCGA dataset. B, mRNA expression comparison between different grade of glioma from TCGA dataset. ***P < .001, ns, not significant

We initially analyzed the mRNA expression profile of E2Fs. As shown in Figure 1A, all E2Fs were generally determined in HGG samples. Compared with LGG, except for E2F3 and E2F5, all E2Fs were highly expressed in HGG samples and correlated with grade progression, with highest expression attributed to glioblastoma (GBM, grade IV) (Figure 1B). To verify our finding, we referred to another dataset, Gravendeel dataset,^26^ which contains 276 cases with corresponding pathology information. The result was similar with TCGA dataset and showed that all E2Fs but E2F5 were elevated in HGG (Figure S1).

### 3.2 Correlation analysis of E2Fs in HGG with critical oncogenes

**Figure 2:**
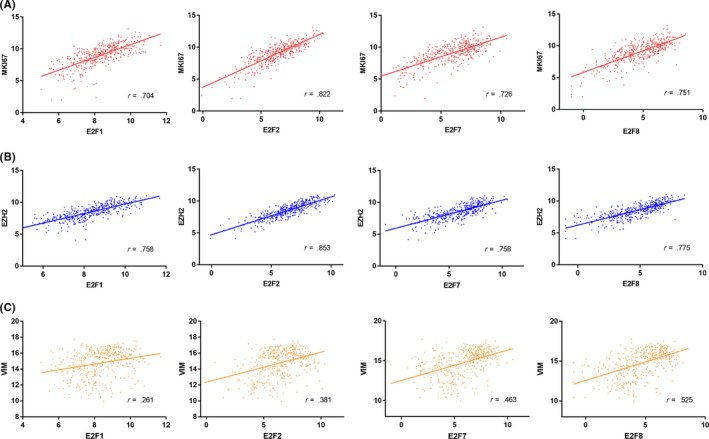
Correlation analysis of E2Fs in HGG with critical oncogenes. A‐C, Correlation analysis of E2Fs with MKI67 (A), EZH2 (B), and VIM (C) in TCGA dataset

As E2Fs were reported to participate in the cell cycle of tumor cells, we next analyzed the association between E2Fs and well‐known cell cycle and proliferation oncogenes (MKI67, HELLS NEK2,^27^ MELK, FOXM1^28^) in HGG. Meanwhile, we selected a variety of widely investigated oncogenes in HGG for further correlation analysis, including stemness associated genes (PROM1,^29^ CD44,^30^ NANOG,^31^ Olig2,^32^ and L1CAM^33^) and therapeutic resistance genes (EZH2,^34^ EGFR,^35^ VIM,^36^ CHEK1,^37^ and AURKA^38^). The unsupervised clustering analysis showed that the correlation signature of E2F1, E2F2, E2F7, and E2F8 were quite similar with each other (Figure S2). Notably, the result demonstrated that E2F1, E2F2, E2F7, and E2F8 were positively correlated with cell cycle and proliferation markers (Figure 2A). However, none of E2Fs were identified to be highly correlated with stemness associated genes. Interesting, we found that E2F1, E2F2, E2F7, and E2F8 were tightly associated with therapeutic resistance genes, including EZH2 (Figure 2B), CHEK1, and AUKRA. In addition, we found E2F7 and E2F8 demonstrated much higher correlation with VIM, a crucial epithelial‐mesenchymal transition (EMT) marker in tumor cells (Figure 2C). All results in this aspect were shown in Table 2. Collectively, the result revealed that higher expression of E2Fs might be responsible for aggressive proliferation and therapeutic resistance of HGG tumor cells.

**Table 2:** Association between E2Fs and well‐known oncogenes in HGG

	Cell cycle					Stemness					Potential therapeutic resistance				
	MKI67	MELK	FOXM1	NEK2	HELLS	PROM1	CD44	NANOG	OLIG2	L1CAM	EZH2	AURKA	CHEK1	VIM	EGFR
E2F1															
*r*	**0.704**	**0.806**	**0.839**	**0.8**	**0.753**	0.189	0.009	0.078	−0.122	−0.125	**0.758**	**0.796**	**0.814**	0.261	0.042
*P* value	<.001	<.001	<.001	<.001	<.001	<.001	0.863	0.122	0.015	0.013	<.001	<.001	<.001	<.001	0.401
E2F2															
*r*	**0.822**	**0.833**	**0.852**	**0.792**	**0.751**	0.241	0.094	0.021	0.049	−0.225	**0.853**	**0.776**	**0.778**	0.381	0.048
*P* value	<.001	<.001	<.001	<.001	<.001	<.001	0.061	0.671	0.334	<.001	<.001	<.001	<.001	<.001	0.339
E2F3															
*r*	0.497	0.333	0.363	0.329	0.435	0.054	−0.142	0.057	0.163	0.1	0.398	0.318	0.337	−0.038	−0.179
*P* value	<.001	<.001	<.001	<.001	<.001	0.281	0.005	0.256	0.001	0.048	<.001	<.001	<.001	0.451	<.001
E2F4															
*r*	0.241	0.409	0.375	0.382	0.337	0.153	0.229	0.081	−0.223	−0.325	0.458	0.463	0.412	0.44	−0.052
*P* value	<.001	<.001	<.001	<.001	<.001	0.002	<.001	0.11	<.001	<.001	<.001	<.001	<.001	<.001	0.302
E2F5															
*r*	0.25	0.346	0.288	0.316	0.321	0.236	0.211	0.026	0.012	−0.265	0.363	0.339	0.338	0.256	0.102
*P* value	<.001	<.001	<.001	<.001	<.001	<.001	<.001	0.607	0.813	<.001	<.001	<.001	<.001	<.001	0.043
E2F6															
*r*	0.231	0.471	0.403	0.481	0.417	0.282	0.258	−0.021	−0.228	−0.378	0.473	0.528	0.508	0.386	0.097
*P* value	<.001	<.001	<.001	<.001	<.001	<.001	<.001	0.683	<.001	<.001	<.001	<.001	<.001	<.001	0.055
E2F7															
*r*	**0.726**	**0.865**	**0.837**	**0.832**	**0.758**	0.294	0.287	0.077	−0.378	−0.321	**0.758**	**0.835**	**0.766**	**0.463**	0.281
*P* value	<.001	<.001	<.001	<.001	<.001	<.001	<.001	0.128	<.001	<.001	<.001	<.001	<.001	<.001	<.001
E2F8															
*r*	**0.751**	**0.882**	**0.84**	**0.831**	**0.732**	0.267	0.336	0.081	−0.345	−0.38	**0.775**	**0.836**	**0.756**	**0.525**	0.234
*P* value	<.001	<.001	<.001	<.001	<.001	<.001	<.001	0.107	<.001	<.001	<.001	<.001	<.001	<.001	<.001

For bold values, the statistically significant *P* values are *P* < .001.

### 3.3 Survival outcome analysis for HGG patients

**Figure 3:**
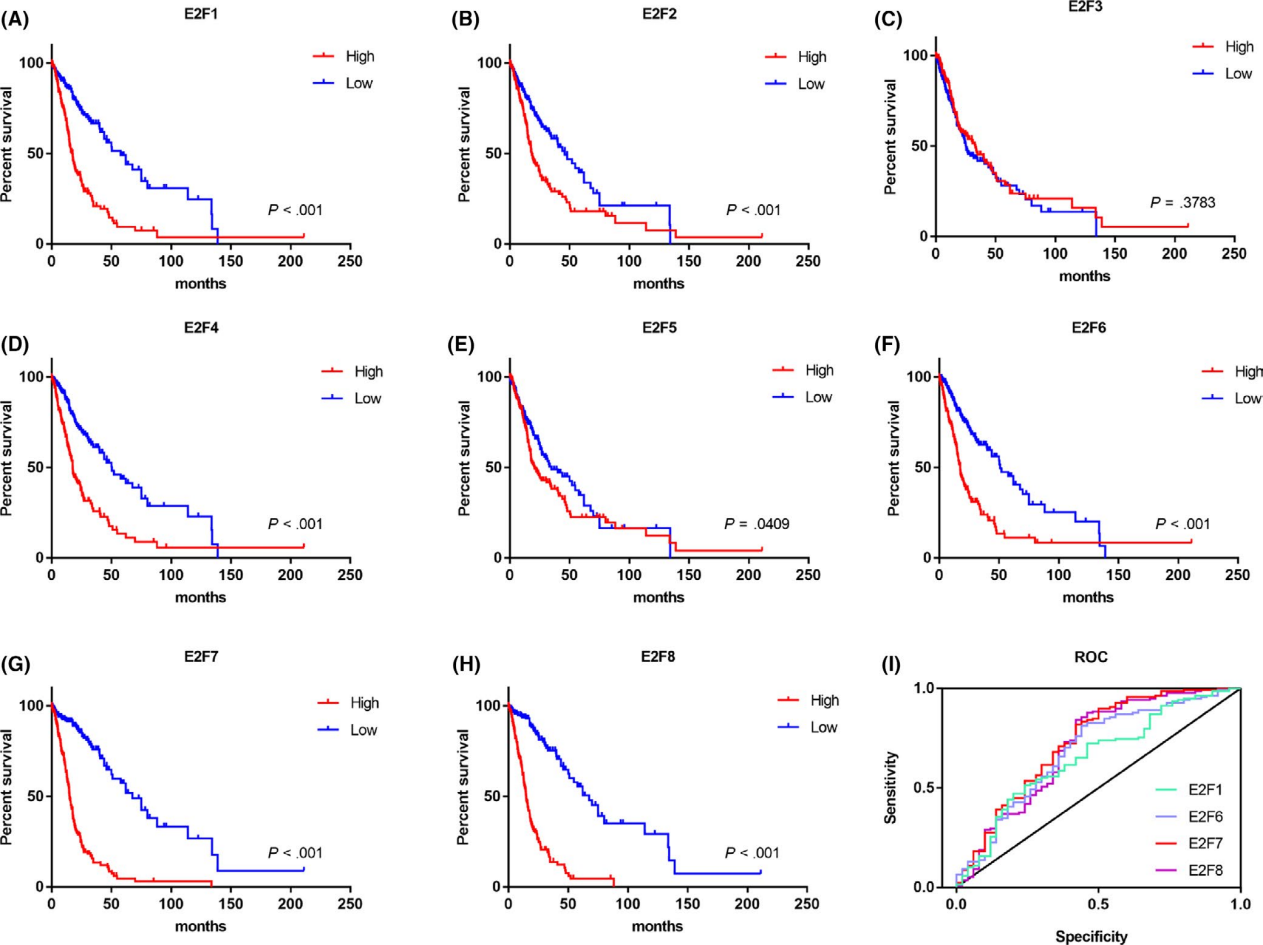
Survival outcome analysis for HGG patients. A‐H, Kaplan‐Meier curve comparing overall survival of HGG patients according to expression of E2Fs in TCGA dataset. Log‐rank test. I, ROC analysis for expression of E2Fs with overall survival in HGG patients. E2F1: AUC = 0.655, *P* = .001, with z test; E2F6: AUC = 0.688, *P* < .001, with z test; E2F7: AUC = 0.731, *P* < .001, with z test; E2F8: AUC = 0.707, *P* < .001, with z test

We next explored the association of E2Fs with patient outcome. A total of 393 patients with overall survival data were enrolled in this analysis. The Kaplan‐Meier (K‐M) analysis result indicated that E2F1, E2F2, E2F4, E2F6, E2F7, and E2F8 were significantly associated with patient overall survival (*P* < .001) (Figure 3A). Interestingly, the median survival demonstrated much bigger difference when analyzed with E2F7 (15 vs 67.5 months) and E2F8 (14.9 vs 67.5 months), compared with other E2Fs. Higher expression of E2F5 was also linked with worse outcome, with relative lower significance (*P* = .0409), while the expression of E2F3 did not show any correlation with patient outcome.

Next, receiver operating characteristic (ROC) curves were constructed to evaluate the area under the curve (AUC) for the potential prediction role of E2Fs (Figure 3I). In total, 188 patients with endpoint data were analyzed and the patients were subgroup by the overall survival. In accordance with the K‐M analysis result, the result of ROC curves showed that E2F1, E2F2, E2F4, E2F6, E2F7, and E2F8 were significant compared with the reference line. Interestingly, E2F7 and E2F8 showed much higher AUC value, 0.731 and 0.707, respectively, compared with E2F1 (0.688), E2F2 (0.604), E2F4 (0.631), and E2F6 (0.655) (Table 3). These data indicate that E2F7 and E2F8 are potential primary candidate of outcome prediction markers for HGG.

**Table 3:** ROC analysis for predicted factors in HGG

Variables	AUC	*P* value
E2F1	0.655	.001
E2F2	0.604	.029
E2F3	0.475	.596
E2F4	0.631	.006
E2F5	0.582	.085
E2F6	0.688	<.001
E2F7	**0.731**	<.001
E2F8	**0.707**	<.001

For bold values, the statistically significant *P* values are *P* < .001.

Given the bigger median survival difference and higher AUC value of E2F7 and E2F8, we further explore their link with clinical signature in HGG. As shown in Figure S3A, higher expression of E2F7 was significantly linked with higher tumor grade (*P* < .001), wild type (WT) of IDH (*P* < .001) and unmethylation status of MGMT promoter (*P* < .001). Interestingly, elevated expression of E2F7 was shown to be associated with older age of HGG patients (*P* < .001). The analysis revealed similar result for E2F8 (Figure S3B). Next, we utilized logistic regression to determine the association of E2F7 or E2F8 expression with prognostic clinicopathologic variables. Analysis (Table 4) demonstrated that elevation of E2F7 was significantly linked with patient age (≤50 vs > 50, OR = 0.183, 95% CI (0.118‐0.284), *P* < .001), tumor grade (IV vs III, OR = 12.792, 95% CI (7.631‐21.444), *P* < .001), mutation status of IDH (WT vs mutant, OR = 29.639, 95% CI (17.111‐51.342), *P* < .001), and methylation status of MGMT promoter (methylation vs unmethylation, OR = 0.126, 95% CI (0.073‐0.215), *P* < .001). In addition, E2F8 demonstrated similar association with these clinicopathologic variables (Table S1). However, no significant differences were identified on sex subgroups. These results showed that E2F7 and E2F8 were closely associated with clinicopathologic features of HGG.

**Table 4:** E2F7 expression associated with pathological characteristics (using logistic regression)

Clinical characteristics	Total number	Odds ratio in E2F7 expression	*P*‐value
Age (≤50 vs > 50)	388	0.183 (0.118‐0.284)	<.001
Sex (male vs female)	388	0.879 (0.585‐1.32)	.534
Grade (IV vs III)	394	12.792 (7.631‐21.444)	<.001
IDH1 status (WT vs mutant)	391	29.639 (17.111‐51.342)	<.001
MGMT promoter status (methylation vs unmethylation)	363	0.126 (0.073‐0.215)	<.001

Lastly, independent prognostic factors were investigated via cox proportional hazard regression models (Table 5). Results of univariate analyses demonstrated that not only E2Fs but also age of patient, IDH mutation status and MGMT promoter methylation status correlated with overall survival. Notably, the multivariate cox regression analyses revealed that E2F7 (HR = 1.818; 95% CI, 1.024‐3.288; *P* = .041) and E2F8 (HR = 1.886; 95% CI, 1.049‐3.390; *P* = .0340) were significant and independent predictors for poor outcome in HGG patients (Table 6). In addition, the multivariate cox regression analyses also revealed that IDH mutation status and patient age were significant and independent predictors. Collectively, the survival analysis revealed the novel role of E2F7 and E2F8 in HGG.

**Table 5:** Univariate Cox regression analyses

Variable	*P* value	HR	95% CI	
			Lower	Upper
E2F1	2.334E‐10	2.924	2.098	4.074
E2F2	6.026E‐04	1.752	1.272	2.414
E2F3	3.586E‐01	0.862	0.628	1.183
E2F4	8.246E‐08	2.430	1.757	3.362
E2F5	1.007E‐01	1.306	0.950	1.796
E2F6	2.935E‐08	2.521	1.818	3.495
E2F7	3.767E‐22	6.269	4.322	9.091
E2F8	6.229E‐21	5.979	4.116	8.685
IDH status	3.958E‐27	8.468	5.744	12.483
MGMT promoter status	3.588E‐11	0.334	0.241	0.462
Age	6.283E‐17	0.199	0.137	0.291
Gender	7.280E‐01	0.945	0.686	1.301

**Table 6:** Multivariate Cox regression analyses

Variable	*P* value	HR	95% CI	
			Lower	Upper
E2F1	1.025E‐01	0.647	0.384	1.091
E2F2	1.618E‐01	1.410	0.871	2.280
E2F3	‐	‐	‐	‐
E2F4	4.184E‐01	1.165	0.805	1.684
E2F5	‐	‐	‐	‐
E2F6	1.736E‐01	0.742	0.483	1.140
E2F7	4.131E‐02	1.818	1.024	3.228
E2F8	3.401E‐02	1.886	1.049	3.390
IDH status	2.808E‐07	4.397	2.499	7.739
MGMT promoter status	3.537E‐01	0.841	0.582	1.213
Age	2.830E‐04	0.432	0.275	0.680
Gender	‐	‐	‐	‐

### 3.4 Bioinformatics analysis of oncogenic role of E2F7 and E2F8 in HGG

**Figure 4:**
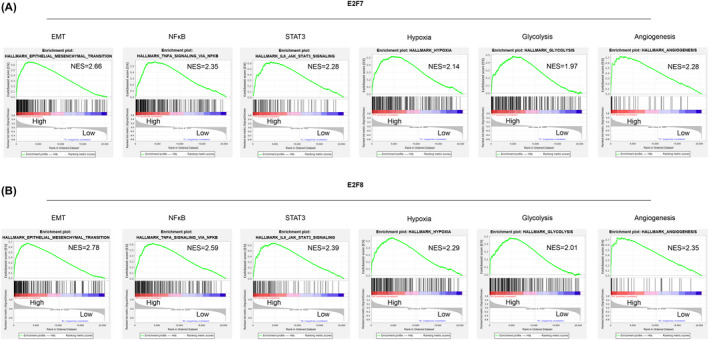
Gene set enrichment analysis for oncogenic role of E2F7 and E2F8 in HGG. A, B, Gene set enrichment analysis (GSEA) of HGG patient samples from TCGA dataset subgrouped by expression of E2F7 (A) and E2F8 (B)

Given the better performance of E2F7 and E2F8 in predicting patient outcome, we further explored the oncogenic role of E2F7 and E2F8 in HGG tumor cells. The gene set enrichment analysis (GSEA) was performed to analyze pathway alterations in E2Fs‐high and E2Fs‐low patient cohorts (subgrouped by median expression of E2F7 or E2F8) (Table S2). As shown in Figure 4, significant differences (normalized *P* < .05, FDR < 0.25) were observed in the enrichment result. Ranked as the top one pathway, epithelial‐mesenchymal transition (EMT) was found to be highly enriched in E2F7 or E2F8 high‐expression group in HGG (Figure 4A,B), a malignant phenotype transition that confers higher radioresistance to tumor cells.^39^ Besides, E2F7 and E2F8 were significantly involved with a variety of signaling pathways promoting tumor initiation and progression in HGG, evidenced by the enrichment of NFκB, STAT3, angiogenesis, hypoxia, and glycolysis pathways in high‐expression phenotype (Figure 4A,B).

### 3.5 E2F7 promotes radioresistance of HGG tumor cells

**Figure 5:**
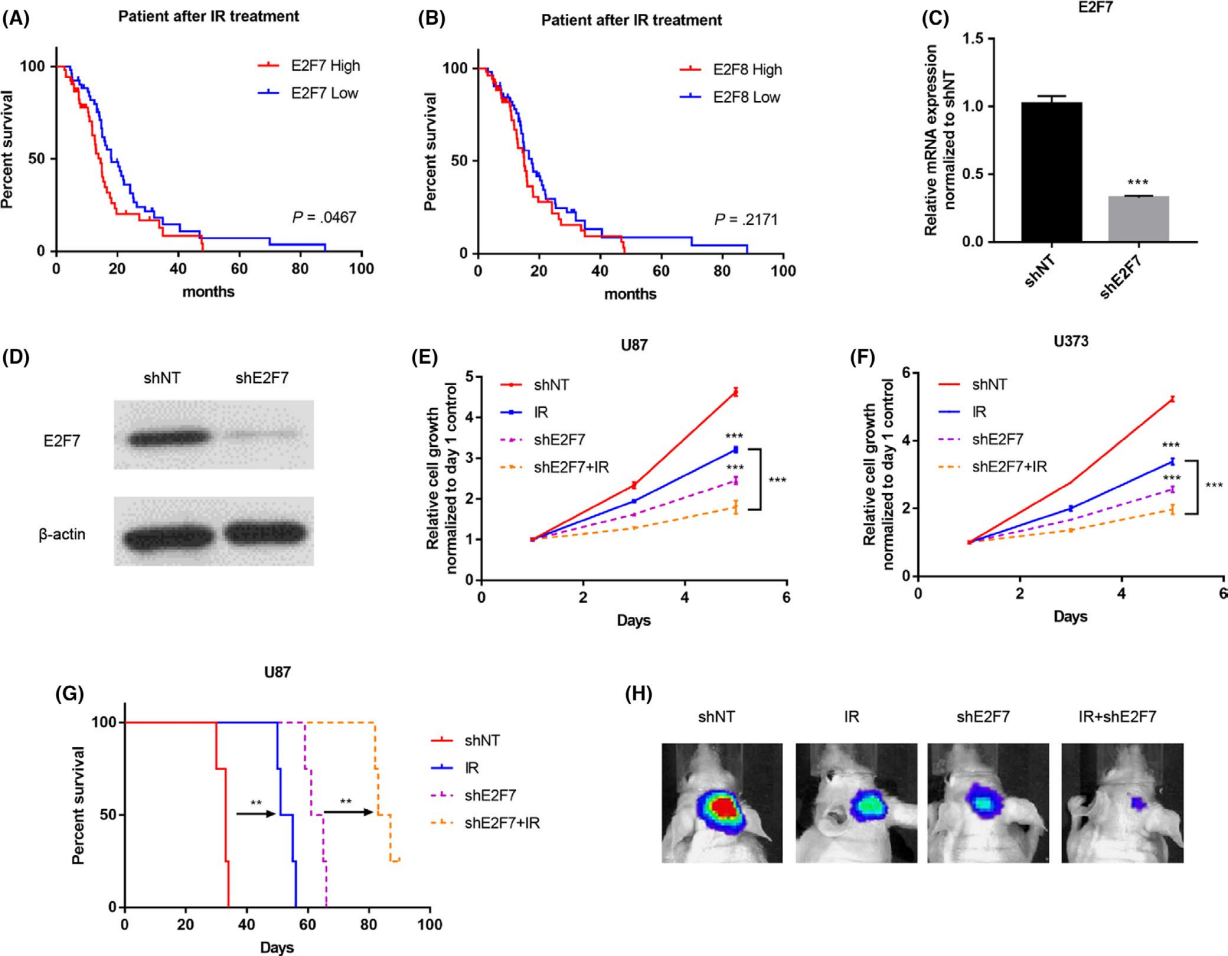
E2F7 promotes radioresistance of HGG tumor cells. A, B, Kaplan‐Meier curve comparing overall survival of HGG patients underwent clinical IR treatment according to expression of E2F7 (A) and E2F8 (B) in TCGA dataset. Log‐rank test. C, qRT‐PCR analysis of E2F7 mRNA expression in U87 cells infected with NT‐shRNA or E2F7‐shRNA. ***P < .001; n = 3. D, WB for E2F7 protein expression in U87 GBM cells treated with NT‐shRNA or E2F8‐shRNA. (E, F) In vitro cell viability assay of U87 (E) and U373 (F) GBM cells treated with/without IR (8 Gy) after pre‐treatment with either NT‐shRNA or E2F7‐shRNA. ***P < .001. G, Kaplan‐Meier curve comparing overall survival of mice intracranially injected with U87 GBM cells pre‐treated with either NT‐shRNA or E2F7‐shRNA, the mice were treated with/without IR (2 Gy for 4 consecutive days) 5 days after tumor cell injection. Log‐rank test. **P < .01. H, Bioluminescence images (BLI) of mice intracranially injected with luciferase‐labeled U373 GBM cells pre‐treated with either NT‐shRNA or E2F7‐shRNA. The mice were treated with IR (2 Gy for 4 consecutive days) 1 week after tumor cell injection

Based on the high correlation with multiple oncogenes (Figure 2) and the enriched oncogenic pathways (Figure 4), we posited that E2F7 or E2F8 might be linked with irradiation therapeutic resistance in HGG, which has been rarely investigated before. Thus, the patients in TCGA dataset underwent clinical treatment (IR or temozolomide (TMZ)) were sorted out and enrolled in the Kaplan‐Meier analysis (subgrouped by median expression of E2F7 or E2F8). The result showed that higher expression of E2F7 was closely associated with worse response to IR treatment (Figure 5A,B). However, the expression of E2F7 and E2F8 did not show significant link with TMZ treatment response (Figure S4A,B). In addition, we found that the expression of E2F7 remarkablely increased after IR treatment (Figure S4C). To further confirm our finding, we utilized lentivirus and infected tumor cells with shRNA either nontargeting control (shNT) or shE2F7. The efficacy of silencing was evaluated via qRT‐PCR and WB, and significant expression reduction of E2F7 was observed in the shE2F7 group (Figure 5C,D). Next, shNT and shE2F7 U87/U373 tumor cells were treated with irradiation treatment. The result of in vitro growth analysis showed that significantly more effective cell killing by irradiation was observed in shE2F7 group (Figure 5E,F). To verify in vivo, the infected U87/U373 tumor cells were injected into brains of nude mouse. The result of survival analysis of mice showed much better outcome in shE2F7 group in the presence of IR treatment (Figure 5G), evidenced by the marked reduction of bioluminescence signal in combination treatment group (Figure 5H). These data further confirmed the role of E2F7 in promoting IR‐resistance of HGG tumor cells.

### 3.6 E2F7 is linked with aggressive oncogenic process and transcriptionally regulates expression of EZH2

**Figure 6:**
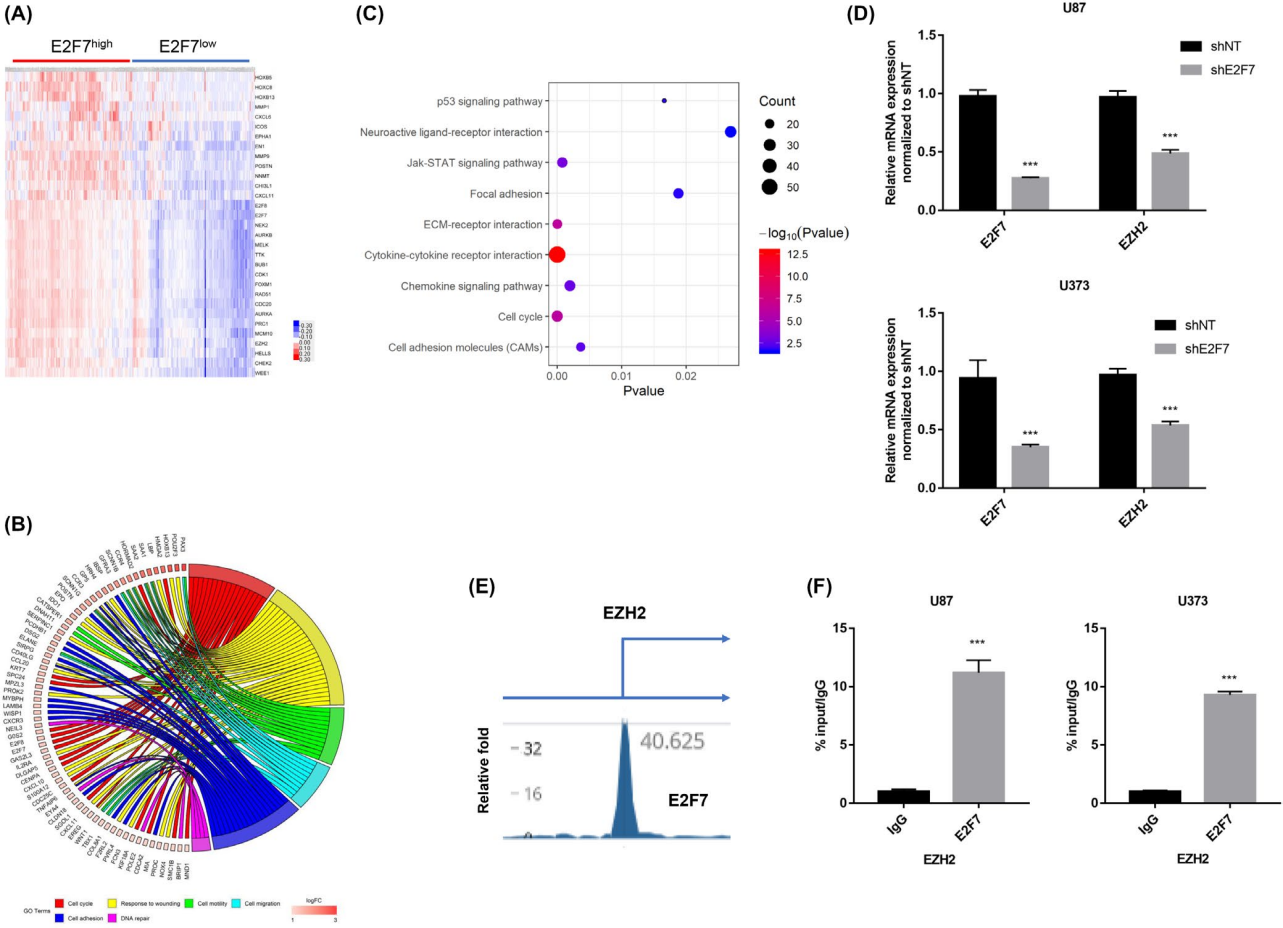
E2F7 is linked with aggressive oncogenic process and transcriptionally regulates expression of EZH2. A, Heatmap depicting the critical DEGs (differential expressed genes) in E2F7‐high and E2F7‐low patient samples from TCGA dataset. B, Gene ontology analysis of DEGs between E2F7‐high and E2F7‐low patient samples from TCGA dataset. C, KEGG (Kyoto Encyclopedia of Genes and Genomes) analysis of DEGs between E2F7‐high and E2F7‐low patient samples from TCGA dataset. D, qRT‐PCR analysis of E2F7 and EZH2 mRNA expression in U87 and U373 tumor cells infected with NT‐shRNA or E2F7‐shRNA. ***P < .001; n = 3. E, Schematic presentation of binding peaks of E2F7 at promoter area of EZH2 in K562 tumor cells. F, ChIP‐PCR analysis showing enrichment of E2F7 at EZH2 promoter in U87 and U373 tumor cells

To determine the potential underline mechanism for the role of E2F7 in promoting radioresistance, we performed additional bioinformatics analysis. Differential expressed genes (DEGs, 2729 genes in total) in TCGA dataset were first identified and selected oncogenes were plotted with heatmap (Figure 6A). Gene ontology analysis showed that E2F7 was highly associated with aggressive cell proliferation, EMT signature (cell migration, adhesion, and motility), and DNA repair (Figure 6B). KEGG analysis further highlighted the critical role of E2F7 in cell proliferation, EMT and revealed a novel role in cellular communications of HGG (Figure 6C). EZH2 has been previously identified as a crucial regulator factor in promoting radioresistance of HGG,^34^ we posited that E2F7 might regulate the transcription activity of EZH2 and promote radioresistance of tumor cells. Via qRT‐PCR, we found that silencing of E2F7 induced significant reduction of EZH2 mRNA expression in U87 and U373 tumor cells (Figure 6D). ChIP‐sequencing data from ENCODE (Encyclopedia of DNA Elements) revealed clear binding peaks for E2F7 at promoter area of EZH2 in K562 tumor cells (Figure 6E). Finally, ChIP‐PCR was performed to determine the occupancy of E2F7 at promoter area of EZH2 and the result showed significant binding pattern of E2F7, compared with IgG (Figure 6F). Collectively, these data suggest E2F7 is involved with multiple oncogenic process and a potentially key regulator of EZH2 transcriptional activity in HGG tumor cells.

## 4 DISCUSSION

Glioma accounts for the vast majority of adult malignant brain tumors, highly infiltrative pattern, aggressive proliferation, therapeutic resistance, and fast recurrence speed as hallmarks.^1^ High‐grade glioma (HGG) demonstrates much worse outcome compared with the low‐grade counterparts. E2Fs play crucial role in aggressive cell cycle, and multiple studies have demonstrated that E2Fs had critical prognostic significance in a variety of cancer types.^16^, ^40^ However, the entirety of the prognostic roles of E2Fs expression in HGG has not yet been systemically investigated.

In this study, the basal expression of E2Fs was firstly explored. The result showed that E2Fs were general expressed in HGG. Interestingly, we found that most of E2Fs showed a grade correlated progression except for E2F3 and E2F5. The correlation analysis of E2Fs with well‐known oncogenes give us a hint on the oncogenic role of individual E2F member. E2F1, E2F2, E2F7, and E2F8 were identified to be highly correlated with cell cycle oncogenes, highlighting their critical role in aggressive proliferation of HGG. However, we did not find high correlation of E2Fs with tumor stemness associated oncogenes. Coincidentally, E2F1, E2F2, E2F7, and E2F8 were top four members that demonstrated significant correlation with potential therapeutic resistance genes. These results revealed that these four E2F members might be critical hub gens for HGG.

The survival analysis further confirmed the prognostic role of E2Fs. Firstly, Kaplan‐Meier analysis showed that, except for E2F3, all other members were significantly linked with worse outcome of HGG patients. The ROC analysis revealed that E2F7 and E2F8 possessed top two highest AUC value in predicting the survival of patients. Most importantly, the multivariate cox regression analyses showed that E2F7 and E2F8 were independent prognostic factors for patient outcome. To our knowledge, E2F1 is widely investigated and confers aggressiveness to HGG, while the role of E2F7 and E2F8 is rarely characterized in HGG. To further understand the oncogenic role of E2F7 and E2F8, we utilized GSEA to identify the pathway alternations based on the expression of these members. To our surprise, E2F7 and E2F8 were found to be highly linked with a variety of oncogenic pathways that were critical in HGG, including NFκB, STAT3, angiogenesis, hypoxia, and glycolysis pathways.

Previous studies have already revealed the role of E2F7 and E2F8 in DNA damage repair process.^23^, ^41^ Given the high correlation with therapeutic resistance‐associated oncogenes and the enrichment of crucial pathways, there is a high possibility that E2F7 and E2F8 are involved in irradiation resistance of HGG. The analysis of patients underwent clinical IR treatment indicated that patients with higher expression of E2F7 showed lower response to IR treatment and suffered faster recurrence. To verify this, we employed lentivirus infection and verify the radioresistance role of E2F7 both in vitro and in vivo. The results showed that in the presence of silencing of E2F7, IR treatment demonstrated more cell‐killing effect. More studies with multiple panel of HGG patients are required to further validate our finding.

For the underlying mechanism of regulation by E2F7 in HGG that confers radioresistance, we believe there are several possible scenarios. First, as an activator transcription factor, E2F7 binds at the corresponding promoter area of target oncogenes and promotes the transcription process. According to the previous studyy,^42^ multiple binding site of E2F7 has been identified in other cancer types. In this study, we confirmed the regulation of EZH2 via ChIP‐PCR analysis. Secondly, E2F7 achieves its function via involving in the crucial pathway activations, indirectly regulating the target oncogenes, like STAT3 pathway as identified via GSEA. Moreover, due to the independence of dimerization proteins, E2F7 serves as a recruitment factor and affects the target gene expression. However, all the above hypotheses warrant further functional validation.

In conclusion, this study systemically analyzed the expression pattern and overall outcome of HGG patients. E2F7 and E2F8 were identified as novel hub genes for aggressiveness of HGG. Pathway alternations highlighted the irradiation protection role of E2F7 and E2F8 in HGG. E2F7 promoted radioresistance of HGG via multiple oncogenic process, including transcriptional regulation of EZH2. Our findings might provide potential experimental evidence for future clinical treatment of HGG.

## CONFLICT OF INTEREST

No conflicts of interest exists in the submission of the manuscript and the manuscript is approved by all authors for publication.
