# Supplementary figures and images for: Expression and prognostic role of E2F transcription factors in high‐grade glioma
Source: CNS Neurosci Ther. 2020 Feb 16;26(7):741–53. doi: 10.1111/cns.13295 (PMC7299000; doi:10.1111/cns.13295)

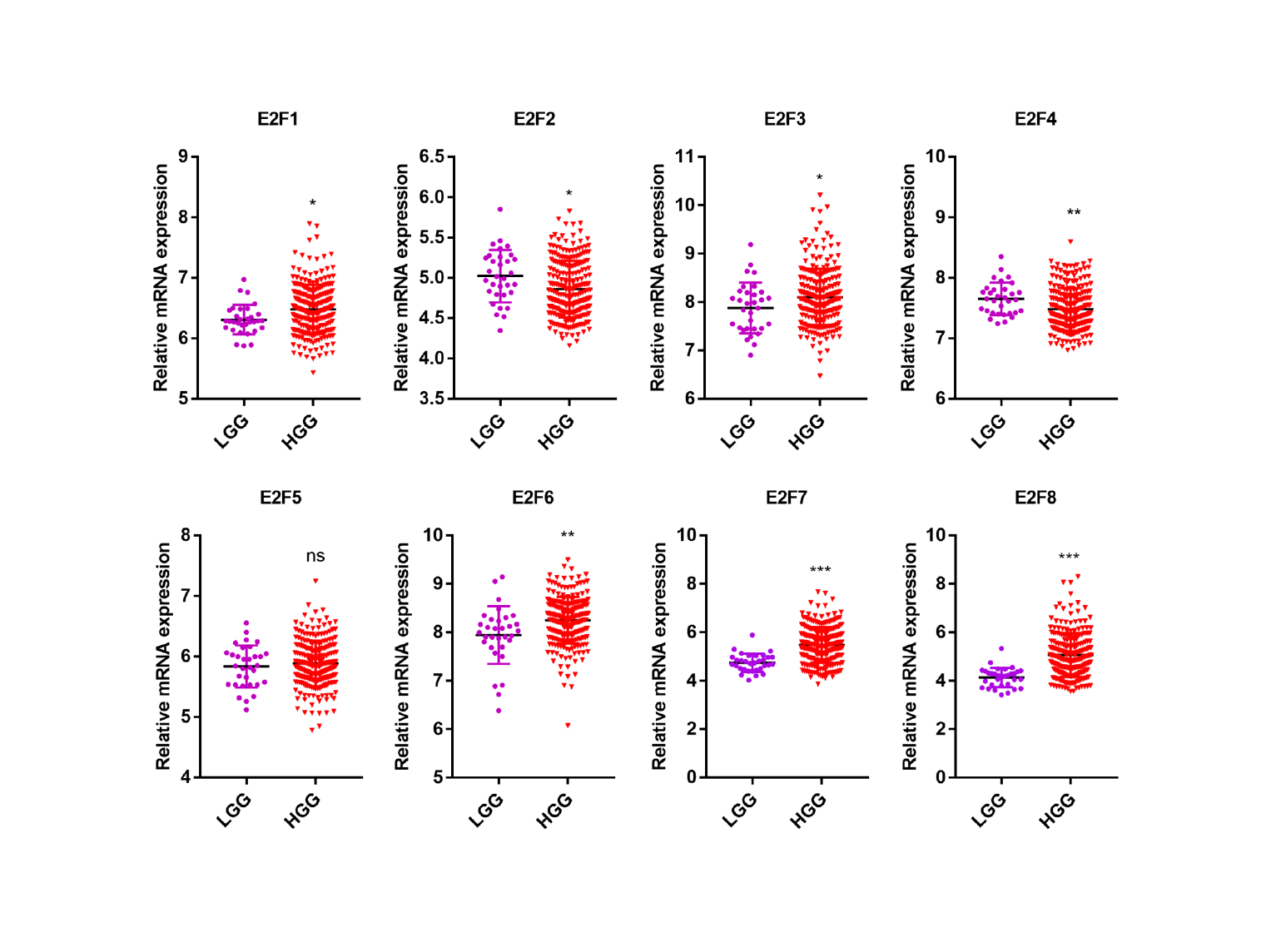

Supplement: Supplementary file 1 [file CNS-26-741-s001.tif]

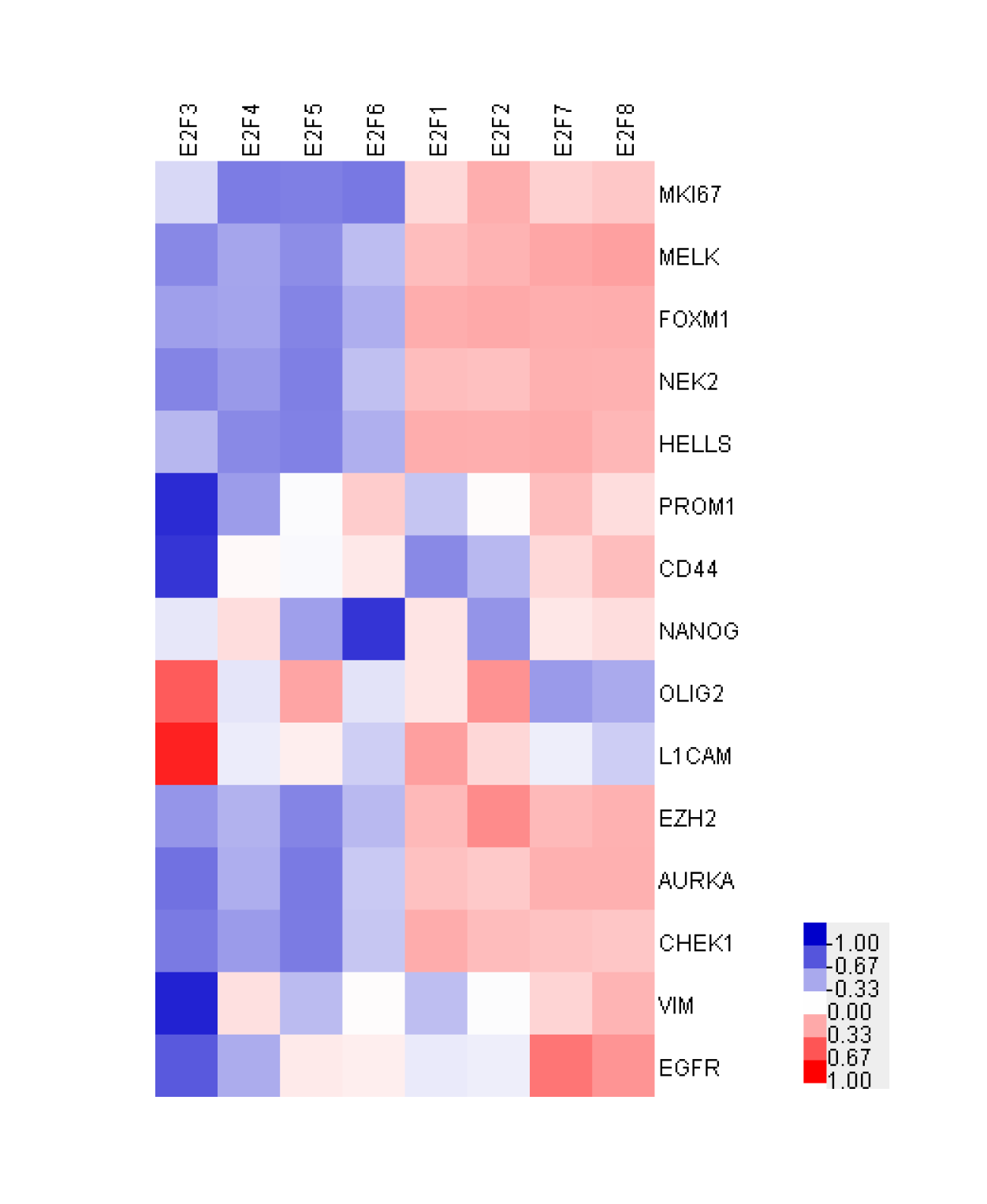

Supplement: Supplementary file 2 [file CNS-26-741-s002.tif]

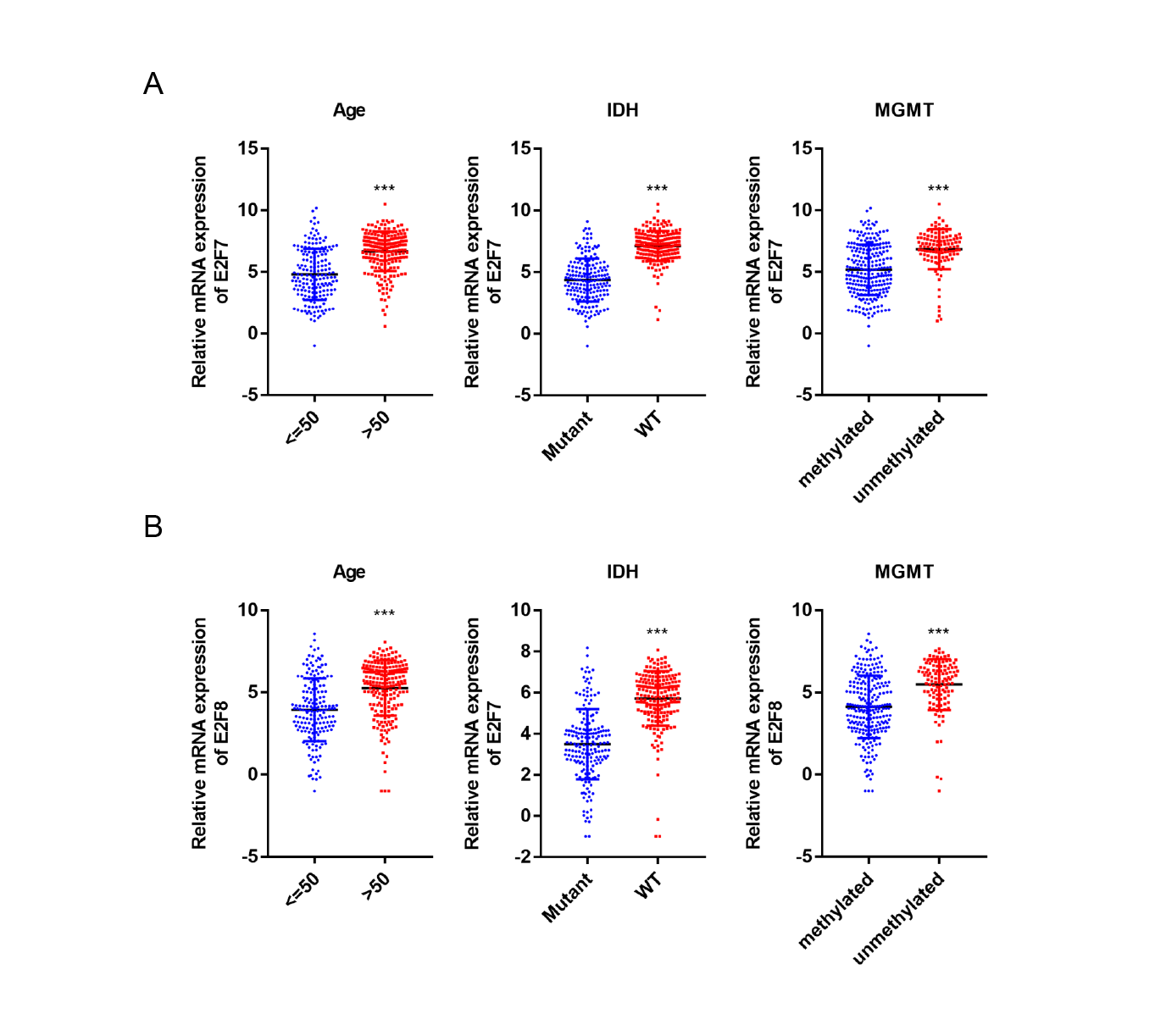

Supplement: Supplementary file 3 [file CNS-26-741-s003.tif]

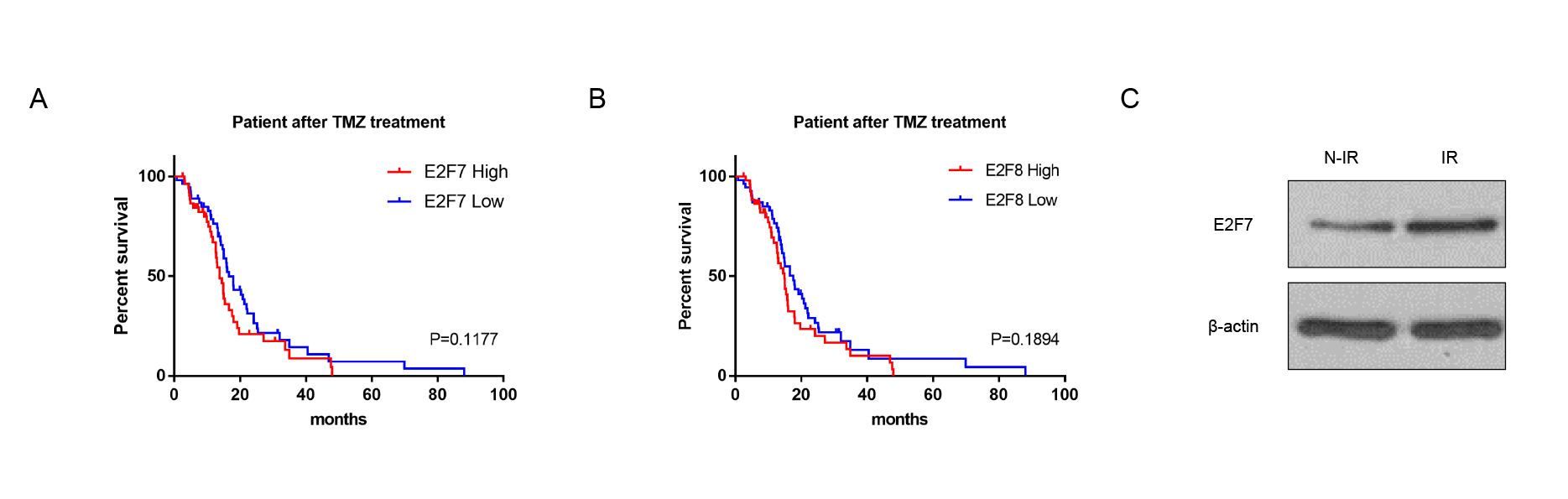

Supplement: Supplementary file 4 [file CNS-26-741-s004.tif]
