# Supplementary material for: Expression and prognostic role of E2F transcription factors in high‐grade glioma
Source: CNS Neurosci Ther. 2020 Feb 16;26(7):741–53. doi: 10.1111/cns.13295 (PMC7299000; doi:10.1111/cns.13295)
Supplement: Supplementary file 5 [file CNS-26-741-s005.docx]

| Table S1 E2F8 expression associated with pathological characteristics (using logistic regression) | | | |
| --- | --- | --- | --- |
| Clinical characteristics | Total number | Odds ratio in E2F8 expression | P‐value |
| Age (≤50 vs >50) | 388 | 0.202 (0.131-0.312) | <0.001 |
| Sex (male vs female) | 388 | 0.917 (0.611-1.378) | 0.678 |
| Grade (IV vs III) | 394 | 17.172 (9.924-29.713) | <0.001 |
| IDH1 status (WT vs mutant) | 391 | 20.5 (12.233-34.355) | <0.001 |
| MGMT promoter status (methylation vs unmethylation) | 363 | 0.179 (0.108-0.297) | <0.001 |
