# Supplementary material for: Expression and prognostic role of E2F transcription factors in high‐grade glioma
Source: CNS Neurosci Ther. 2020 Feb 16;26(7):741–53. doi: 10.1111/cns.13295 (PMC7299000; doi:10.1111/cns.13295)
Supplement: Supplementary file 6 [file CNS-26-741-s006.docx]

| Table S2 Gene sets enriched in high E2F7 expression phenotype | | | |
| --- | --- | --- | --- |
| NAME | NES | NOM p-val | FDR q-val |
| HALLMARK_EPITHELIAL_MESENCHYMAL_TRANSITION | 2.6637092 | 0 | 0 |
| HALLMARK_E2F_TARGETS | 2.623721 | 0 | 0 |
| HALLMARK_INTERFERON_GAMMA_RESPONSE | 2.614363 | 0 | 0 |
| HALLMARK_G2M_CHECKPOINT | 2.5904675 | 0 | 0 |
| HALLMARK_ALLOGRAFT_REJECTION | 2.511699 | 0 | 0 |
| HALLMARK_INFLAMMATORY_RESPONSE | 2.3863032 | 0 | 0 |
| HALLMARK_INTERFERON_ALPHA_RESPONSE | 2.3550332 | 0 | 0 |
| HALLMARK_TNFA_SIGNALING_VIA_NFKB | 2.34781 | 0 | 0 |
| HALLMARK_ANGIOGENESIS | 2.2784877 | 0 | 0 |
| HALLMARK_IL6_JAK_STAT3_SIGNALING | 2.2750878 | 0 | 0 |
| HALLMARK_COAGULATION | 2.1745558 | 0 | 0 |
| HALLMARK_HYPOXIA | 2.1363194 | 0 | 0 |
| HALLMARK_APOPTOSIS | 2.0832336 | 0 | 0 |
| HALLMARK_IL2_STAT5_SIGNALING | 2.0776792 | 0 | 0 |
| HALLMARK_COMPLEMENT | 2.062908 | 0 | 0 |
| HALLMARK_KRAS_SIGNALING_UP | 1.9984474 | 0 | 8.21E-05 |
| HALLMARK_GLYCOLYSIS | 1.972271 | 0 | 7.73E-05 |
| HALLMARK_MTORC1_SIGNALING | 1.939811 | 0 | 7.30E-05 |
| HALLMARK_DNA_REPAIR | 1.8401692 | 0 | 3.46E-04 |
| HALLMARK_MYC_TARGETS_V1 | 1.8150072 | 0 | 4.03E-04 |
