# Supplementary material for: Expression and prognostic role of E2F transcription factors in high‐grade glioma
Source: CNS Neurosci Ther. 2020 Feb 16;26(7):741–53. doi: 10.1111/cns.13295 (PMC7299000; doi:10.1111/cns.13295)
Supplement: Supplementary file 7 [file CNS-26-741-s007.docx]

| Table S3 Gene sets enriched in high E2F8 expression phenotype | | | |
| --- | --- | --- | --- |
| NAME | NES | NOM p-val | FDR q-val |
| HALLMARK_EPITHELIAL_MESENCHYMAL_TRANSITION | 2.7775888 | 0 | 0 |
| HALLMARK_INTERFERON_GAMMA_RESPONSE | 2.7589934 | 0 | 0 |
| HALLMARK_E2F_TARGETS | 2.6871924 | 0 | 0 |
| HALLMARK_ALLOGRAFT_REJECTION | 2.6304963 | 0 | 0 |
| HALLMARK_G2M_CHECKPOINT | 2.6060147 | 0 | 0 |
| HALLMARK_TNFA_SIGNALING_VIA_NFKB | 2.590617 | 0 | 0 |
| HALLMARK_INFLAMMATORY_RESPONSE | 2.5168428 | 0 | 0 |
| HALLMARK_INTERFERON_ALPHA_RESPONSE | 2.4781675 | 0 | 0 |
| HALLMARK_IL6_JAK_STAT3_SIGNALING | 2.388429 | 0 | 0 |
| HALLMARK_ANGIOGENESIS | 2.3464787 | 0 | 0 |
| HALLMARK_HYPOXIA | 2.290177 | 0 | 0 |
| HALLMARK_COAGULATION | 2.2551868 | 0 | 0 |
| HALLMARK_IL2_STAT5_SIGNALING | 2.171811 | 0 | 0 |
| HALLMARK_APOPTOSIS | 2.1701124 | 0 | 0 |
| HALLMARK_COMPLEMENT | 2.109606 | 0 | 0 |
| HALLMARK_KRAS_SIGNALING_UP | 2.086679 | 0 | 0 |
| HALLMARK_GLYCOLYSIS | 2.0094383 | 0 | 0 |
| HALLMARK_MTORC1_SIGNALING | 1.9932703 | 0 | 0 |
| HALLMARK_MYC_TARGETS_V1 | 1.9042938 | 0 | 0 |
| HALLMARK_DNA_REPAIR | 1.8552272 | 0 | 3.41E-04 |
